# Protein-controlled versus restricted protein versus low protein diets in managing patients with non-dialysis chronic kidney disease: a single centre experience in Australia

**DOI:** 10.1186/s12882-016-0341-4

**Published:** 2016-09-13

**Authors:** Maria Chan

**Affiliations:** 1 Department of Nutrition and Dietetics, The St. George Hospital, Gray Street, Kogarah, NSW 2217 Australia; 2 Department of Renal Medicine The St. George Hospital, Kogarah, NSW 2217 Australia; 3 Department of Nutrition and Dietetics, School of Medicine and Illawarra Health and Medical Research Institute, University of Wollongong, Wollongong, NSW 2522 Australia; 4 St. George Clinical School, School of Medicine, The University of New South Wales, Sydney, NSW 2217 Australia

## Abstract

Nutrition has been an important part of medical management in patients with chronic kidney disease for more than a century. Since the 1970s, due to technological advances in renal replacement therapy (RRT) such as dialysis and transplantation, the importance of nutrition intervention in non-dialysis stages has diminished. In addition, it appears that there is a lack of high-level evidence to support the use of diet therapy, in particular the use of low protein diets to slow down disease progression. However, nutrition abnormalities are known to emerge well before dialysis is required and are associated with poor outcomes post-commencing dialysis. To improve clinical outcomes it is prudent to incorporate practice research and quality audits into routine care, as part of the continuous clinical practice improvement process. This article summarises the experience of and current practices in a metropolitan tertiary teaching hospital in Sydney, Australia.

## Background

The main goals of nutritional management of patients in end stage kidney disease (ESKD) are to maintain optimal nutrition status and prevent complications associated with deteriorating renal function, such as high blood pressure, malnutrition, symptom burden, electrolytes/fluid imbalances, and increased cardiovascular risk. The additional specific goals for non-dialysis CKD stages 4–5 patients are to (1) preserve renal function and delay disease progression, (2) delay onset of uraemic symptoms, (3) have a healthy start of dialysis, and improve the quality of life of patients, especially if they are on a conservative pathway. From the author’s personal experience, referrals by nephrologists to dietitians for non-dialysis CKD nutrition intervention have dropped significantly since the release of the Modification of Diet in Renal Disease (MDRD) [1] study in 1994 as the initial results did not show a significant reduction in the rate of renal disease progression. To date, referral remains ad hoc depending on the subjective belief of the nephrologists, despite the supportive evidence available in the literature [2–4], MDRD follow-up study [5], Cochrane systematic review [6], and renal nutrition practice guidelines, e.g. Kidney Disease Outcome Quality Initiative (KDOQI)D [7] and Dietitians Association of Australia (DAA) [8]. This has been a common phenomenon in the renal community in Australia for many years. It was highlighted in a recent national survey of nutrition practice in stages 4–5 non-dialysis CKD that showed approximately 17 % and 46 % of renal dietitians perceived their nephrologists/renal team as “very supportive’ or “somewhat supportive” respectively of the use of low protein diets [9]. The majority of respondents (34.2 %) reported less than 10 % of patients received structured nutrition intervention before starting dialysis in their centres. However, referral for dietary management to optimise blood pressure, body weight/energy balance, fluid, serum potassium, and phosphate imbalances could be more regular. Furthermore, due to an exponential increase in dialysis patients over the last two decades and inadequate renal dietitian staffing, services have been shifted to focus on patients on dialysis and transplantation programs instead. In coming years, the role of nutrition intervention in non-dialysis CKD could become important again, as there is ample evidence suggesting pre-dialysis nutrition factors are associated with dialysis outcomes [10, 11], as well as a growing trend for supporting patients on conservative care pathways [12, 13].

### Research leading to current practices

As recommended by the KDOQI and DAA guidelines for haemodialysis patients, all hospital and satellite centre patients receive six-monthly nutrition reviews. Prevalence of malnutrition as rated by subjective global assessment (SGA) was consistently high at 40-50 % in reviews performed in the early 2000s, and many of these patients were new on the dialysis program. Further study indicated that less than 30 % of new patients received nutrition intervention in pre-dialysis stages. Together with education needs for other disciplines, a multidisciplinary pre-dialysis assessment clinic was established to provide timely management for patients on a pre-dialysis pathway. Nephrologists refer patients with GFR <30 mL/min to this renal specialty clinic for assessment and education by the clinical nurse consultant, social worker, pharmacist, and a dietitian in a session lastings approximately 3 h. Patients and caretakers are informed about “Renal Options” or different treatment choices such as haemodialysis, continuous ambulatory peritoneal dialysis (CAPD) verse automated peritoneal dialysis (APD), conservative care and transplantation, whereas the social worker assessed their psycho-social status and needs [14]. The renal dietitian became responsible for the nutrition assessment (Table 1) and intervention under “blanket referral” based on clinical practice guidelines and agreed department protocols. This means, by protocols, there is no screening or triaging by the nurse consultant, and all patients are assessed by the dietitian. After the initial assessment, patients are informed of their results and receive brief education on the role of dietary management in CKD, in particular, the concept of how nutrition intervention helps to preserve renal function, reduce complications, maintain better nutritional status, and to aim for a healthy start of dialysis. In general, patients are advised to attend ongoing counselling and intervention sessions in the Nutrition and Dietetics Department until dialysis starts. If patients wish to make a decision regarding the uptake of intervention, they are advised to contact the detitians when ready or the nephrologist to re-refer, if any nutritional issues arise. In addition, nephrologists also refer other non-dialysis CKD stages 3b-5, including conservative care patients directly to renal dietitians for nutrition counselling. As part of the clinical practice improvement process, the roles of nutrition intervention and practices were revisited in the non-dialysis CKD group in our centre, addressing the question, “Is nutrition management good enough only when it starts at or near dialysis initiation?”

**Table 1:** Nutrition management protocols of CKD Stages 4–5 (non- dialysed)

Dietary Protocol: In General	As per clinical practice guidelines and a balanced diet
Protein	Approximately 0.75-1.0 g /kg IBW/d (Australian RDI) Approximately 70 % HBV protein Remark: ▪ ~0.6 g/kg IBW/d (and no less) for patients with severe symptoms (usually applicable to patients in advance stage of conservative care) ▪ For nutrition support or repletion ~ 1.0 g /kg IBW/d ▪ A high protein diet for nutrition support in malnourished patients, or weight reduction in overweight/obese patients is inappropriate
Energy	Aim to attain and maintain IBW Depending on physical activity level 35–45 Kcal (150-190KJ)/kg IBW/d for <60 years 30–35 Kcal (130-150KJ)/kg IBW/d for >60 years ▪ energy from CHO approximately 50-60 % ▪ energy from Fat approximately 30-35 %. Adapted to individual needs in the case of under-nutrition or overweight/obesity
Sodium	If hypertension or oedema present: Approximately 80 mmol/d (no added salt) ▪ May need lower sodium intake if severe oedema present, e.g. 50 mmol/d ▪ May need higher sodium intake in patients with salt-losing nephropathy
Potassium	No restriction unless hyperkalaemia present 40-70 mmol/d if restriction required
Phosphorus	<1000 mg/d if hyperphosphatemia present + phosphate binders
Fat	▪ Encouraged Mono- and poly-unsaturated fats ▪ Saturated fat <10 % of energy ▪ Cholesterol <300 mg/d
Alcohol	No more than 2 standard drinks per day or advised by renal physician
Vitamins & Minerals (diet)	Near RDI levels
Vitamins & Minerals (supplementation)	May need individualised calcium, iron and vitamin D supplementation. May need supplementation of Vitamin B complex, Vitamin C and folate acid near RDI levels if protein intake is <60 g/day
Fluid	UO + 500 ml/d, depending on balance
Dietary Pattern	Regular inclusion of fruit and vegetables, and dietary fibre
Recommended intervention (outpatient, minimum)	
Initial appointment ~ 2 h, then review every 1–3 months, and more frequently if clinically indicated. Then 6 monthly in stable patients (minimum 6 h per annum). • Stable CKD and pre-dialysis patients: ▪ Follow up until dialysis commences • Conservative pathway: ▪ Follow up until withdrawing from treatment or for end of life care	

Modified from the “Nutrition Protocols for the Management of People with Kidney Disease, The St. George Hospital, Sydney [17]. Abbreviations: IBW, ideal body weight; RDI, recommended daily intake; HBV, high biological value

In a ten-year clinical cohort study of patients who commenced planned dialysis programs, the prevalence of malnutrition as scored by SGA B & C was found to be approximately 52 %. Multivariate analysis indicated malnutrition was an independent predictor of mortality, irrespective of the glomerular filtration rate (eGFR) at which dialysis started; and high body mass index (BMI) values did not show any protective effects [11]. In addition, the combination of malnutrition (SGA B & C) and overweight/obesity (BMI ≥ 26 kg/m^2^) was associated with the worse outcomes. From experience, nutritional status improves in most patients after starting dialysis; unfortunately the moderate to severely malnourished patients often showed sub-optimal improvement despite intense nutritional support. In the study of nutritional characteristics of patients attending the pre-dialysis clinic, mean eGFR was 17.3 ± 6.5 mL/min/1.73 m^2^ and 40.5 % of patients were rated as malnourished. Indeed, nutrition abnormalities merge well before dialysis is needed [15]. Factors associated with abnormal nutrition parameters included declining eGFR, symptom burden causing poor intake, poor habitual eating habits, and inappropriate intake due to lack of nutrition knowledge in managing CKD. “Is nutrition management good enough only when it starts at or near dialysis initiation?” The answer is no. The results of these studies suggested structured nutrition management should be implemented well before dialysis is required, and even before the pre-dialysis stage to improve health outcomes, as well as potential healthcare cost savings. Furthermore, not all ESKD patients benefit from dialysis, therefore timely nutrition intervention is vital to preserve renal function and maintain quality of life in patients choosing conservative care.

### Current practices

In our centre, renal dietitians receive referrals from nephrologists, either directly or through “blanket referral” in the multidisciplinary pre-dialysis assessment and renal supportive care clinics. Patients receive intervention within the framework of the nutrition care process (NCP), namely structured care including assessment, diagnosis, intervention, and monitoring/evaluation [16]; as well as dietary prescription and frequency and duration of intervention as recommended by the clinical and work practice guidelines summarised into the agreed local protocols [17]. Table 1 summaries the nutrition management protocols for CKD (non-dialysis) stage 4–5. These protocols are regularly updated with agreements sought from the renal team during the review process.

“Low protein diet” has not been well accepted by the Australian nephrology community, as there is no high-level evidence to support such practice. Many clinicians perceive a “low protein diet” or “restricted protein diet” as restrictive and leading to treatment burden and malnutrition. With these beliefs, a “free diet” is often thought to help improve nutrition status and quality of life. Unfortunately, it doesn’t guarantee appropriate and adequate intake, or good nutritional status. Problems can range from very poor spontaneous intake due to uraemia, or on the other hand, excessive habitual protein intake leads to uraemic toxin build-up and exacerbates symptoms [18]. In addition, protein foods are naturally high in acids, purine, phosphorous and potassium. Therefore, uncontrolled protein food intake may also lead to other complications such as greater acid load, hyperuricaemia, hyperphosphatemia and hyperkalaemia. The national dietary survey reported that the habitual protein intake of the average Australian adult is almost twice the RDI level of 0.75 g/kg/d [19–21]. Patients often fail to recognise symptoms and the gradual reduction of food intake leads to deteriorating nutritional status. These nutritional problems surfaced during the period of decline in referral for nutrition intervention in non-dialysis CKD stages.

Uraemia is a word derived from two ancient Greek words, *ouron* (urine) and *haima* (blood), to describe the presence of increased urea and other nitrogenous end products of protein and amino acid metabolism in blood [22, 23]. Patients are in a chronic stage of protein intolerance or protein waste intoxication. To strike the balance between avoiding a build-up of nitrogenous waste and preventing protein energy wasting [24], patients must consume the right amount of protein with adequate energy. However, the ideal level of protein intake for patients in stages 4–5 CKD is controversial [25].

When energy intake is adequate, the physiological requirement of protein is approximately 0.6 g/kg ideal body weight (IBW)/d [26, 27]. The recommended daily intake (RDI) for adults in the general Australian population is approximately 0.75 g/kg/d (0.75 g/kg/d for women and 0.84 g/kg/d for men) [27]. Clinically stable non-dialysed CKD patients have similar physiological requirements of ~0.6 g/kg IBW/d when energy ≥25 kcal/kg/d is ingested, and even more stable with 30–35 kcal/kg/d [28], which are the recommended levels in clinical practice [7]. In the elderly, protein RDI is approximately 1.0 g/kg/d [27]. In view of these considerations, our patients are counselled on a “protein controlled diet” of 0.75-1.0 g/kg/d with adequate energy to attain or maintain ideal body weight. This practice is further supported by the results of a randomised controlled trial offering individualised nutrition counselling [8] with a protein prescription of 0.75-1.0 g/kg/d, which showed significant improvements in nutritional status and symptom scores when compared to the control [29]. Malnourished patients requiring nutrition support are advised ~1.0 g/kg IBW/d for repletion and anabolism; whereas stable conservatively managed patients with severe symptoms may require ~0.6 g/kg IBW/d and no less. To maintain good quality of life when patients are approaching end of life care, it is important to remind them about appropriate eating to alleviate symptoms and gain strength. Counselling will help them make informed decisions as to whether or not to follow any recommendations. High protein diets are not recommended for CKD patients, as they are associated with faster progression rate [30, 31]. Unfortunately, in recent years high protein diets for weight loss have become fashionable in the overweight/obese population, including CKD patients before they have a chance to learn about CKD nutrition.

The use of a very low protein diet, VLPD (0.3 g/kg/d plus keto-analogues of amino acids) in CKD patients is well studied and demonstrated favourable outcomes [32–34]. VLPD was trialled in Australia in the 1980s. Despite the favourable results of slowing down disease progression rate and symptom control, such treatment has never been adopted beyond the clinical trial stage. Currently, these products are not available in Australia and the VLPD regimens are not currently included in our nutrition management protocols Table 1. Protein requirements and other nutrition considerations in CKD stages 1–3 have been reviewed and published recently [35, 36]. Again, protein intake near the RDI level is recommended. In summary, our nutrition management protocols adopt recommendations of our national [8] and international [7, 33] guidelines.

Regular nutrition assessment, evaluation, and monitoring are vital to track patients’ progress and outcomes. Table 2 shows the nutrition and clinical assessment checklist used, including the repeated measure in subsequent follow-up visit to monitor progress and diet adherence. It is important to focus on patient-centred outcomes; in addition to assessing the clinical parameters, patients and caretakers are encourage to inform dietitians of the enablers and barriers to better diet adherence.

**Table 2:** Nutrition and clinical assessment checklist

Nutritional assessment		Demographic	
(A) Anthropometry^a^			Age
	Weight and weight history		Gender
	Height		Race
	Body Mass Index		Social: occupation, living arrangement
	Triceps skinfold	Clinical data	
	Mid-Arm circumference		Cause of kidney failure
(B) Biochemistry /blood results^a^			Co-morbidities, presence of:
	Serum creatinine, eGFR		Coronary artery disease
	Serum albumin, potassium, phosphate and C reactive protein, CRP (if available) Haemoglobin		Chronic lung disease
(C) Clinical signs and symptoms^a^			Cerebral vascular disease
	Appetite score		Peripheral vascular disease
	Presence of nausea		Diabetes Mellitus
	Presence of taste change		Other conditions affecting nutrition status e.g. cancer, liver disease etc.
	Presence of other symptoms (see section “O”)		Smoking habits
(D) Dietary intake/ Drug^a^		Future treatment option	
	Diet history (structured diet history method)		Conservative care
	Nutrient & food group analyses		Haemodialysis(home/hospital)
	Drug (relevant medications e.g., phosphate binders etc. and drug- nutrient interaction)		Peritoneal dialysis
(E) Exercise and Physical activity^a^			Transplantation
(F) Functional status^a^			
	Handgrip strength	Discharge from current program	
(O) Others^a^			Date
	Subjective Global Assessment, SGA (7 point scale)		Treatment modality or other reasons
	Palliative care outcome scale (POS)		eGFR and nutritional status
	How patients are feeling? Any question about the diet? Barrier and enabler to better diet adherence.		

Remark: recommended frequency and duration of intervention (1) initial assessment and education - 2 h (can be over 1–3 sessions depending on the patient’s understand and skill to adhere to the diet, (2) then minimum 6 h per annum in established patients. Reference: in member only section of Dietitians Association of Australia, “Workforce recommendations for Renal dietitians in Australia and New Zealand” produced by the Australian and New Zealand renal dietitians workforce planning group, updated 2016

^a^Repeat measure in subsequent follow-up visit to monitor progress

### Beyond the protein-controlled diet in CKD

The risk affecting renal disease progression is multi-factorial and complex. In addition to traditional dietary modifications of energy and nutrients, e.g. protein, sodium, potassium, phosphorous, fluid, and fats [37], there is growing evidence to address the benefits of other food components and dietary patterns for kidney health. These include the alkali-inducing effect of fruit and vegetable consumption in decreasing markers of kidney injury [38–41]. A high dietary fibre intake has been associated with reduced risk of inflammation and mortality in patients with CKD [42, 43] and the potential benefit of probiotics to decrease uremic toxin production [44–46]. Dietary patterns, such as a Mediterranean diet are associated with lower mortality risk in CKD [47]; and a randomised control trail showed promising results in improving dyslipidaemia, markers of inflammation, and lipid peroxidation in stages 1–3 CKD patients [48]. Campared to a Western dietary pattern, the Dietary Approach to Stop Hypertension (DASH)-style diet appeared to protect against rapid eGFR decline [49]. In fact, many of these recommendations are in line with national dietary guideline for healthy eating for adults [50, 51]. Therefore, CKD diets actually encourage healthy food intake.

### Practical issues

Nutrition in CKD is a therapeutic intervention, as it is an integral part of medical care.

However, nutrition interventions are often seen as “lifestyle modifications”, therefore clinicians and/or patients see them as low priority or optional treatments, especially when dietary modifications are mistakenly associated with restriction and treatment burden.

Renal dietitians counsel patients and caretakers according to the dietary prescription as detailed in Table 1, and the information is tailor - made to suit their levels of comprehension and education. To improve adherence through coaching for better knowledge, skill and motivation, the main emphasis in the implementation steps are:

- Dietary practice
  - ▪ To tailor the diet plan.
  - ▪ To inform the quality and quantity of foods, and assist with pictures and drawings to reduce the burden of measurement or weighing.
  - ▪ To provide practical tips, e.g. shopping lists, recipes, etc.
- Build positive thinking mind-set
  - ▪ The diet should be practical and prescriptive, not restrictive.
  - ▪ The specific quality and quantity of foods recommended help to set realistic goals, so patients understand what and how much to eat.
  - ▪ The diet is similar to the recommendations for all Australians. It is a healthy diet for good health and the kidneys (i.e. no deprivation).
  - ▪ For the protein-controlled diet, eat enough protein, “not too much and not too little”, as the kidneys have limited ability to remove waste, etc.
  - ▪ Symptomatic conservative care patients often experience “food phobia”. A planned diet helps set realistic eating goals of knowing to eat adequately without exacerbating symptoms.
  - ▪ Provide essential information to help patients make informed decisions as to whether or not to follow the recommendations.
- Clear and realistic goal setting, especially around patient-centred outcomes

### Challenges and strategies to achieve better outcomes

A multi-disciplinary approach to nutrition management is vital to improve patient care [52]. Together with healthcare professionals, patients and their caretakers are regarded as part of the renal team and are involved in decision-making and the planning of treatments.

There are many challenges to address. A change of diet is a change of habits; and patients’ acceptance of change and intervention is vital, as is their willingness to attend clinics. One of the major complaints is not being able to attend too many medical appointments due to failing health or inconvenience. On the other hand, consistent with the literature [53, 54], many patients wish to include dietary guidance and intervention to prevent disease progression. Most importantly, common goals and a belief in nutrition therapy among the renal team allows timely structured intervention by the dietitians for delaying the need of and a healthier start of dialysis with a better 12 month survival [55, 56]. When the data of our centre is analysed, it will be interesting to compare results in different settings. The baseline nutritional status varied widely in our population [15], ranging from underweight and malnourished to well-nourished with morbidly obese, as well as sarcopenic obesity. At this stage, through intervention, we have observed improvement of body weight, handgrip strength and SGA score in many malnourished patients, and gradual planned weight loss in overweight patients, as a result, a number of them required a lesser dose of antihypertensive medications. On the other hand, a small but significant number of patients declined or dropped out from nutrition intervention due to various reasons that are to be investigated. Examples are the burden to attend too many medical appointments, parking cost and a lack of understanding in the diet therapy etc. A detailed evaluation of all patients’ progress and outcomes since the inception of this clinic is currently underway, the results will help gain more insight into the reality of nutrition intervention in such setting.

Regular communications, including patient meetings, reporting, quality audits, and ongoing research are importing strategies to improve care.

## Conclusion

Optimal protein nutrition is part of the multifaceted nutritional therapy approach to managing patients with non-dialysis CKD, irrespective of the terminology used, i.e. “protein-controlled” versus “restricted protein” versus “low protein” diets. Appropriate protein prescription partners with adequate energy, other nutrients, food components and dietary patterns to form effective therapeutic interventions.

## References

[1] Klahr S, Levey AS, Beck GJ, Caggiula AW, Hunsicker L, Kusek JW, Striker G (1994). The effects of dietary protein restriction and blood-pressure control on the progression of chronic renal disease. Modification of Diet in Renal Disease Study Group. N Engl J Med.

[2] Bergstrom J (1984). Discovery and rediscovery of low protein diet. Clin Nephrol.

[3] Mitch WE, Remuzzi G (2004). Diets For Patients With Chronic Kidney Disease, Still Worth Prescribing. J Am Soc Nephrol.

[4] Walser M, Mitch WE, Maroni BJ, Kopple JD (1999). Should protein intake be restricted in predialysis patients?. Kidney Int.

[5] Levey AS, Greene T, Beck GJ, Caggiula AW, Kusek JW, Hunsicker LG, Klahr S (1999). Dietary protein restriction and the progression of chronic renal disease: what have all of the results of the MDRD study shown? Modification of Diet in Renal Disease Study group. J Am Soc Nephrol.

[6] Fouque D, Laville M, Boissel JP (2006). Low protein diets for chronic kidney disease in non diabetic adults. Cochrane Database Syst Rev.

[7] K/DOQI Clinical Practice Guidelines for Nutrition in Chronic Renal Failure, Kidney Disease Outcome Quality Initiative (NKF KDOQI)™, The National Kidney Foundation [http://www2.kidney.org/professionals/KDOQI/guidelines_nutrition/doqi_nut.html]. Accessed Jan 2016.

[8] Ash S, Campbell K, MacLaughlin H, McCoy E, Chan M, Anderson K, Corke K, Dumont R, Lloyd L, Meade A (2006). Evidence based practice guidelines for the nutritional management of chronic kidney disease. Nutr. Diet..

[9] Chan M, Nageswaran M, Patwardhan A, RYAN C, STEVENSON J, PROBST Y. Perceived nutrition management priorities and current practice in progressive chronic kidney disease stages 4–5 In: *51st Annual* Scientific Meeting of the Australian and New Zealand Society of Nephrology*.* Nephrol. 2015;20(Supp.3):23–59

[10] Stenvinkel P, Barany P, Chung SH, Lindholm B, Heimburger O (2002). A comparative analysis of nutritional parameters as predictors of outcome in male and female ESRD patients. Nephrol Dial Transplant.

[11] Chan M, Kelly J, Batterham M, Tapsell L (2012). Malnutrition (Subjective Global Assessment) Scores and Serum Albumin Levels, but not Body Mass Index Values, at Initiation of Dialysis are Independent Predictors of Mortality: A 10-Year Clinical Cohort Study. J Ren Nutr.

[12] O’Connor NR, Kumar P (2012). Conservative management of end-stage renal disease without dialysis: a systematic review. J Palliat Med.

[13] Brown MA, Collett GK, Josland EA, Foote C, Li Q, Brennan FP (2015). CKD in elderly patients managed without dialysis: survival, symptoms, and quality of life. Clin J Am Soc Nephrol.

[14] Chan M, Brown M. Nutrition management in pre-dialysis assessment clinic – 18 months experience. In: Australian and New Zealand Society of Nephrology, 40th Annual Scientific Meeting Nephrology vol. 9 (Suppl.): Nephrology; 2004: A1–A43

[15] Chan M, Kelly J, Batterham M, Tapsell L (2014). A high prevalence of abnormal nutrition parameters found in predialysis end-stage kidney disease: is it a result of uremia or poor eating habits?. J Ren Nutr.

[16] Nutrition Care Process, Academy of Nutrition and Dietetics, USA [http://www.eatrightpro.org/resources/practice/nutrition-care-process]. Accessed Jan 2016.

[17] Nutrition Protocols for the Management of People with Kidney Disease, The St. George and Sutherland Hospitals, Departments of Renal Medicine/Nutrition and Dietetics [https://stgrenal.org.au/sites/default/files/upload/Dietitian/Renal_Nutrition_Management_Protocol_2015.pdf]. Accessed Jan 2016.

[18] Mandayam S, Mitch WE (2006). Dietary protein restriction benefits patients with chronic kidney disease. Nephrology (Carlton).

[19] National Nutrition Survey: nutrient intakes and physical measurements. Cat. no. 4805.0. Australian Bureau of Statistics, Canberra, Australia [www.abs.gov.au/ausstats/abs@.nsf/Lookup/95E87FE64B144FA3CA2568A9001393C0]. Accessed Jan 2016.

[20] Dietary Guidelines for Australian Adults, background information page 6–7. National Health and Medical Research Council (NHMRC), Australian Govenment [http://www.nhmrc.gov.au/_files_nhmrc/file/publications/synopses/n33.pdf]. Accessed Jan 2016.

[21] Protein, Australian Health Survey: Updated Results, 2011–2012. Australian Bureau of Statistics, Canberra, Australia [http://www.abs.gov.au/ausstats/abs@.nsf/Lookup/by%20Subject/4364.0.55.007 ~ 2011-12 ~ Main%20Features ~ Protein ~ 706]. Accessed Jan 2016.

[22] Bergstrom J. Chapter 5 Uremic Toxicity. In: Kopple JD, Massry SG, editors. Management of Renal Disease, First Edition. Baltimore: William & Wilkins; 1997.

[23] The Freedictionary [http://www.dictionary.com/browse/uremia]. Accessed Jan 2016.

[24] Fouque D, Kalantar-Zadeh K, Kopple J, Cano N, Chauveau P, Cuppari L, Franch H, Guarnieri G, Ikizler TA, Kaysen G (2008). A proposed nomenclature and diagnostic criteria for protein-energy wasting in acute and chronic kidney disease. Kidney Int.

[25] Piccoli GB, Vigotti FN, Leone F, Capizzi I, Daidola G, Cabiddu G, Avagnina P (2015). Low-protein diets in CKD: how can we achieve them? A narrative, pragmatic review. Clin Kidney J.

[26] FAO/WHO/UNU (1985). Energy and protein requirements. Technical Report Series 724.

[27] Dietary Protein. Nutrient Reference Values (NRV) for Australia and New Zealand. Australian government. National Health and Research Council (NMMRC) [http://www.nrv.gov.au/nutrients/protein.htm]. Accessed Jan 2016.

[28] Kopple JD, Monteon FJ, Shaib JK (1986). Effect of energy intake on nitrogen metabolism in nondialyzed patients with chronic renal failure. Kidney Int.

[29] Campbell KL, Ash S, Bauer JD (2008). The impact of nutrition intervention on quality of life in pre-dialysis chronic kidney disease patients. Clin Nutrition (Edinburgh, Scotland).

[30] Knight EL, Stampfer MJ, Hankinson SE, Spiegelman D, Curhan GC (2003). The impact of protein intake on renal function decline in women with normal renal function or mild renal insufficiency. Ann Intern Med.

[31] Friedman AN (2004). High-protein diets: potential effects on the kidney in renal health and disease. Am J Kidney Dis.

[32] Brunori G, Viola BF, Parrinello G, De Biase V, Como G, Franco V, Garibotto G, Zubani R, Cancarini GC (2007). Efficacy and safety of a very-low-protein diet when postponing dialysis in the elderly: a prospective randomized multicenter controlled study. Am J Kidney Dis.

[33] Aparicio M, Bellizzi V, Chauveau P, Cupisti A, Ecder T, Fouque D, Garneata L, Lin S, Mitch WE, Teplan V (2012). Keto Acid Therapy in Predialysis Chronic Kidney Disease Patients: Final Consensus. J Renal Nutri.

[34] Aparicio M, Bellizzi V, Chauveau P, Cupisti A, Ecder T, Fouque D, Garneata L, Lin S, Mitch WE, Teplan V (2012). Protein-Restricted Diets Plus Keto/Amino Acids - A Valid Therapeutic Approach for Chronic Kidney Disease Patients. J Renal Nutri.

[35] Modification of lifestyle and nutrition interventions for management of early chronic kidney disease, KHA-CARI guidelines (Kidney Health Australia-Caring for Australisians with Renal Impairment) [http://www.cari.org.au/CKD/CKD%20early/Modification_of_Llifestyle_Nutrition_ECKD.pdf]. Accessed Jan 2016.

[36] Johnson DW, Atai E, Chan M, Phoon RKS, Scott C, Toussaint ND, Turner GL, Usherwood T, Wiggins KJ (2013). KHA-CARI Guideline: Early chronic kidney disease: Detection, prevention and management. Nephrol.

[37] Textbook of Nutritional Management of Renal Disease. Third Edition. In: Kopple JD, Massry SG, Kalanter-Zadeh K, editors. Academic Press, Elsevier Publishing; 2013.

[38] Goraya N, Simoni J, Jo C, Wesson DE (2012). Dietary acid reduction with fruits and vegetables or bicarbonate attenuates kidney injury in patients with a moderately reduced glomerular filtration rate due to hypertensive nephropathy. Kidney Int.

[39] Uribarri J, Oh MS (2012). The key to halting progression of CKD might be in the produce market, not in the pharmacy. Kidney Int.

[40] Goraya N, Simoni J, Jo CH, Wesson DE (2013). A comparison of treating metabolic acidosis in CKD stage 4 hypertensive kidney disease with fruits and vegetables or sodium bicarbonate. Clin J Am Soc Nephrol.

[41] Goraya N, Simoni J, Jo CH, Wesson DE (2014). Treatment of metabolic acidosis in patients with stage 3 chronic kidney disease with fruits and vegetables or oral bicarbonate reduces urine angiotensinogen and preserves glomerular filtration rate. Kidney Int.

[42] Krishnamurthy VM, Wei G, Baird BC, Murtaugh M, Chonchol MB, Raphael KL, Greene T, Beddhu S (2012). High dietary fiber intake is associated with decreased inflammation and all-cause mortality in patients with chronic kidney disease. Kidney Int.

[43] Evenepoel P, Meijers BK (2012). Dietary fiber and protein: nutritional therapy in chronic kidney disease and beyond. Kidney Int.

[44] Koppe L, Mafra D, Fouque D (2015). Probiotics and chronic kidney disease. Kidney Int.

[45] Rossi M, Johnson DW, Campbell KL (2015). The Kidney-Gut Axis: Implications for Nutrition Care. J Ren Nutr.

[46] Rossi M, Johnson DW, Morrison M, Pascoe EM, Coombes JS, Forbes JM, Szeto C-C, McWhinney BC, Ungerer JPJ, Campbell KL. Synbiotics Easing Renal Failure by Improving Gut Microbiology (SYNERGY): A Randomized Trial. Clin J Am Soc Nephrol. 2016;11(2):223–31. 10.2215/CJN.05240515 PMC4741035 26772193

[47] Huang X, Jimenez-Moleon JJ, Lindholm B, Cederholm T, Arnlov J, Riserus U, Sjogren P, Carrero JJ (2013). Mediterranean diet, kidney function, and mortality in men with CKD. Clin J Am Soc Nephrol.

[48] Mekki K, Bouzidi-bekada N, Kaddous A, Bouchenak M (2010). Mediterranean diet improves dyslipidemia and biomarkers in chronic renal failure patients. Food Funct.

[49] Lin J, Fung TT, Hu FB, Curhan GC (2011). Association of dietary patterns with albuminuria and kidney function decline in older white women: a subgroup analysis from the Nurses’ Health Study. Am J Kidney Dis.

[50] Eat for Health, Australian Dietary Guidelines. National Health and Research Council (NH&MRC), Australian Government [https://www.nhmrc.gov.au/_files_nhmrc/publications/attachments/n55_australian_dietary_guidelines_130530.pdf]. Accessed Jan 2016.

[51] 2015–2020 Dietary Guidelines for Americans, Center for Nutrition Policy and Promotion, United States Department of Agriculture (USDA) [http://www.cnpp.usda.gov/2015-2020-dietary-guidelines-americans]. Accessed Jan 2016.

[52] Multidisciplinary or multifaceted renal care in early chronic kidney disease, KHA-CARI guidelines (Kidney Health Australia-Caring for Australisians with Renal Impairment) [http://www.cari.org.au/CKD/CKD%20early/Modification_of_Llifestyle_Nutrition_ECKD.pdf]. Accessed Jan 2016.

[53] Tong A, Sainsbury P, Chadban S, Walker RG, Harris DC, Carter SM, Hall B, Hawley C, Craig JC (2009). Patients’ Experiences and Perspectives of Living With CKD. Am J Kidney Dis.

[54] Palmer SC, Hanson CS, Craig JC, Strippoli GF, Ruospo M, Campbell K, Johnson DW, Tong A (2015). Dietary and fluid restrictions in CKD: a thematic synthesis of patient views from qualitative studies. Am J Kidney Dis.

[55] Slinin Y, Guo H, Gilbertson DT, Mau LW, Ensrud K, Collins AJ, Ishani A (2011). Prehemodialysis care by dietitians and first-year mortality after initiation of hemodialysis. Am J Kidney Dis.

[56] Westland GJ, Grootendorst DC, Halbesma N, Dekker FW, Verburgh CA (2015). The nutritional status of patients starting specialized predialysis care. J Ren Nutr.

